# Impact of deranged B cell subsets distribution in the development of HCV-related cirrhosis and HCC in type two diabetes mellitus

**DOI:** 10.1038/s41598-020-77416-0

**Published:** 2020-11-23

**Authors:** Fadwa A. Abdelwahab, Khaled M. Hassanein, Helal F. Hetta, Mohamed O. Abdelmalek, Asmaa M. Zahran, Omnia El-Badawy

**Affiliations:** 1 Assiut Health Administration, Assiut, Egypt; 2 Medical Microbiology and Immunology Department, Faculty of Medicine, Assiut University, Assiut, 71515 Egypt; 3 Department of Internal Medicine, College of Medicine, University of Cincinnati, Cincinnati, OH 45267-0595 USA; 4 Tropical Medicine and Gastroenterology Department, Faculty of Medicine, Assiut University, Assiut, Egypt; 5 Department of Clinical Pathology, South Egypt Cancer Institute, Assiut University, Assiut, Egypt

**Keywords:** Immunology, Applied immunology, Lymphocytes

## Abstract

Type II diabetes (T2D) may worsen the course of hepatitis C virus infection with a greater risk of liver cirrhosis (LC) and hepatocellular carcinoma (HCC). In chronic viral infections, the deranged B cell subset signifies uncontrolled disease. The study aimed to verify the relation between B cell subsets’ distribution and liver disease progression in chronic hepatitis C (CHC) patients with T2D. A total of 67 CHC patients were divided into two groups; 33 non-diabetic and 34 with T2D. Each group was subdivided into CHC-without LC or HCC (N-CHC), CHC-with LC (CHC-LC), and CHC-with HCC (CHC-HCC). Twenty-seven healthy individuals also participated as controls. Flow cytometry was used to analyze CD19^+^ B cell subsets based on the expression of CD24 and CD38. CD19^+^CD24^hi^CD38^hi^ Immature/transitional B cells elevated in diabetic than non-diabetic patients. In diabetic patients, while CD19^+^CD24^+^CD38^−^ primarily memory B cells were higher in CHC-N and CHC-HCC groups than LC with a good predictive accuracy of LC, the opposite was observed for CD19^+^CD24^−^CD38^−^ new memory B cells. Only in diabetic patients, the CD19^+^CD24^int^CD38^int^ naïve mature B cells were high in CHC-HCC patients with good prognostic accuracy of HCC. Merely in diabetic patients, several correlations were observed between B cell subsets and liver function. Immature/transitional B cells increase remarkably in diabetic CHCpatients and might have a role in liver disease progression. Memory and Naïve B cells are good potential predictors of LC and HCCin diabetic CHCpatients, respectively. Further studies are needed to investigate the role of the CD19^+^CD24^−^CD38^−^ new memory B cells in disease progression in CHC patients.

## Introduction

Hepatitis C virus (HCV) is the leading cause of chronic liver disease that can eventually lead to liver cirrhosis (LC) and hepatocellular carcinoma (HCC)^1^. Type 2 diabetes (T2D) is the most familiar form of diabetes, accounting for above 90% of all diabetic patients^2^. T2D is a well-known cofactor that may worsen the outcomes of HCV infection. It is associated with a greater risk of cirrhosis and HCC development, entailing poor prognosis in patients with and without cirrhosis^3,4^. Hereafter, studying the immunopathogenesis and looking for new and specific immunological markers to predict the progression of chronic hepatitis C (CHC) to LC and HCC in T2D patients has become of great scientific interest.

Generally, B cells play a central role in obesity, insulin resistance, and glucose intolerance. They promote inflammation by activating proinflammatory T cells and secretion of proinflammatory cytokines, including IL-6 and TNF-α^5^. B cells may induce insulin resistance by the activation of Th1 and CD8^+^ T cells and the release of pathogenic IgG antibodies^6,7^. These pathogenic antibodies aggravate the metabolic disease^8^ via activation of immune cells, mostly Fc-dependent activation of macrophages inducing secretion of proinflammatory cytokines^7^. Thus, B cells are prospective promising therapeutic targets for T2D^5^.

Five main B cell subsets can be identified in the peripheral blood based on the expression of the two developmental regulation markers CD24 and CD38, in conjunction with the B cell lineage marker CD19. Based on these surface markers, B cell populations include the immature/transitional B cells (CD19^+^CD24^hi^CD38^hi^), naïve mature B cells which have not encountered an antigen (CD19^+^CD24^int^CD38^int^), antibody-secreting plasmablasts (CD19^+^CD24^−^CD38^+^), primarily memory B cells (CD19^+^CD24^+^CD38^−^) and new memory (CD19^+^CD24^−^CD38^−^)^9–12^_._

Inconsistent data exists on the phenotype of regulatory B cells (Bregs), immune regulation by B cells is most likely not restricted to a specific B-cell subset, and virtually all human B cells can produce IL-10^13^. The highest percentage of Bregs secreting IL-10 was reported within the CD24^hi^CD38^hi^ immature/transitional B cells^14,15^. Furthermore, immune regulation by CD24^hi^CD38^hi^ B cells was associated with promising clinical outcome in patients with chronic inflammatory diseases^16^, and IL-10 producing Bregs have been proposed to have a role in the pathogenesis of hepatitis B virus infection^17^.

In chronic viral infections as human immunodeficiency virus (HIV), deranged B cell functions and subset distribution signifies uncontrolled disease^18^. The levels of immature transitional B cells were shown to be related to some conditions with humoral immune deficiency, including HIV infection^19^. HCV infection is associated with increased B cell activation and proliferation with increased risk of autoimmune and B cell lymphoproliferative disorders^20–24^. Few studies have investigated the alteration in B cell subsets in patients with HCV^25,26^, and the role of the different B‐cell subsets in chronic HCV infection is incompletely understood. To date, as far as we know, no one has addressed the relations of B cell subsets with the liver disease progression in CHC patients with T2D. Thus, the study aimed to verify the possibility of an association between the different B cell subsets distribution and liver disease progression in CHC patients with T2D.

## Materials and methods

### Study Subjects

A total of 67 CHC patients presented to Al Rajhi Liver Hospital, Assiut, Egypt (tertiary care university hospital) were enrolled in this case–control study. Twenty-seven age and sex-matched healthy individuals also participated as controls. We excluded patients treated with interferon, ribavirin, or immunomodulating agents, co-infected with hepatitis A, B viruses, or human immunodeficiency virus, those having alcohol or drug-induced liver diseases, or those with positive anti-schistosomal antibodies. The Ethics Committee of the Faculty of Medicine, Assiut University, reviewed and accepted the research proposal (IRB no. 17100797). All methods were performed in full accordance with the regulations of Assiut University Faculty of Medicine. All participants provided informed consent before sharing in the study.

Patients were divided into two main groups; 33 non-diabetic CHC patients and 34 CHC patients with T2D. Each group was further subdivided according to the stage of liver disease into three sub-groups; CHC-without LC or HCC (N-CHC), CHC-with LC (CHC-LC), and CHC-with HCC (CHC-HCC). CHC patients were diagnosed by fluctuations of serum transaminases levels for more than six months, positive HCV antibodies, and serum HCV-RNA. The diagnosis of LC was based on clinical, biochemical, and ultrasonographic findings. The diagnosis of HCC was established by the typical imaging criteria in abdominal triphasic computed tomography or abdominal dynamic MRI with diffusion with or without raised serum alpha-fetoprotein.

Diabetes mellitus type II was identified by finding: fasting blood glucose (FBG) ≥ 126 mg/dL (7 mmol/L); (fasting is defined as no caloric intake for at least 8 h), Hemoglobin A1c (HbA1c) ≥ 6.5%, Two-hour plasma glucose level ≥ 200 mg/dL (11.1 mmol/L) during a 75-g oral glucose tolerance test or random plasma glucose ≥ 200 mg/dL (11.1 mmol/L) in a patient with classic hyperglycemia symptoms (polyuria, polydipsia, polyphagia, and weight loss) or hyperglycemic crisis^27^.

### Flow cytometry analysis

Flow cytometry was accomplished by fluorescence-activated cell sorter (FACS Calibur, Becton Dickinson, USA) using a set of fluorochrome-labeled monoclonal antibodies against B cell surface markers (APC-conjugated anti-CD19, FITC-conjugated anti-CD24, and PE-conjugated anti-CD38). All monoclonal antibodies were obtained from R&D Systems, USA.

**Figure 1:**
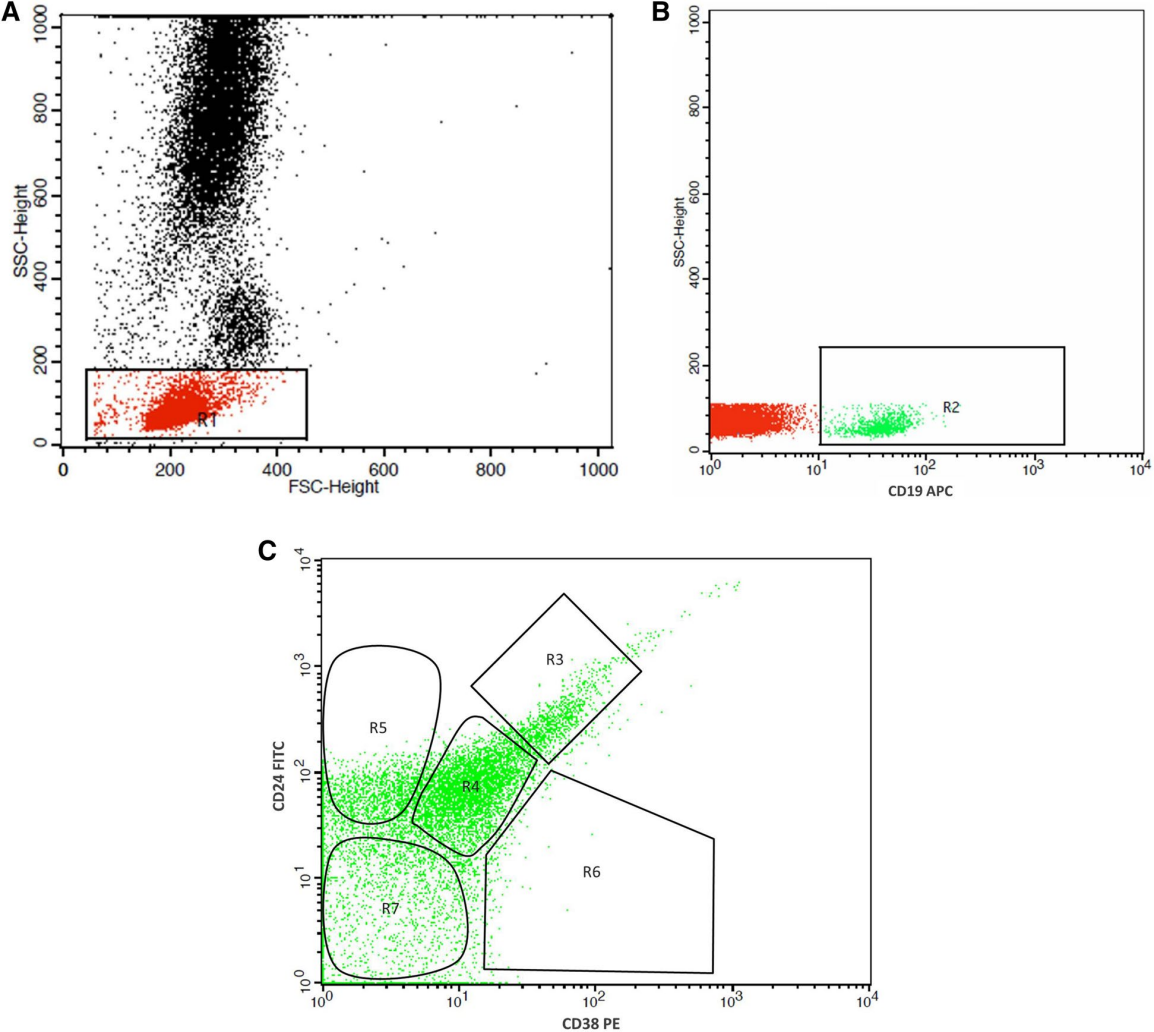
Flow cytometry analysis of B cell subsets. **A** The lymphocyte population was defined within the PBMCs on the forward and side scatter plot by drawing (R1). **B** The expression of CD19 on lymphocytes was identified, then CD19^+^ cells were gated through (R2) for further analysis of CD24 and CD38. **C** The percentages of B cell subsets [CD24^hi^CD38^hi^, CD24^int^CD38^int^, CD24^+^CD38^−^, CD24^−^CD38^+^ and CD24^−^CD38^−^ B cells] were calculated by drawing the regions (R3), (R4), (R5), (R6) and (R7), respectively.

One hundred microliters of heparinized blood were transferred into a clean tube, and RBCs lysing solution was added. The pellet after centrifugation was washed with phosphate-buffered saline (PBS). Samples were centrifuged again, and ten microliters of each of the monoclonal antibodies (CD19, CD 24, and CD38) were added to the pellet. The mixture was incubated for 15 min at 4 °C in the dark. Appropriate isotype controls were processed in a parallel manner without adding B cell monoclonal antibodies. A minimum of 50,000 events was collected for each sample analysis. Cell Quest software (BD, USA) was used for flow cytometric analysis. Through the forward scatter (FSC)/side scatter (SSC) dot plot, the PBMCs population was selected to analyze CD19 expression. After that, the CD19^+^ B cells were selected for further analysis of B cell subsets based on the expression of CD24 and CD38. CD19^+^CD24^hi^CD38^hi^ were considered as immature/transitional B cells , CD19^+^CD24^int^CD38^int^ are naïve mature B cells, CD19^+^CD24^+^CD38^−^ are primarily memory B cells, CD19^+^CD24^−^CD38^+^ are plasmablasts and CD19^+^CD24^−^CD38^−^ are new memory B cells. The different B cell subsets were expressed as percentages within the CD19^+^ lymphocytes (Fig. 1).

**Figure 2:**
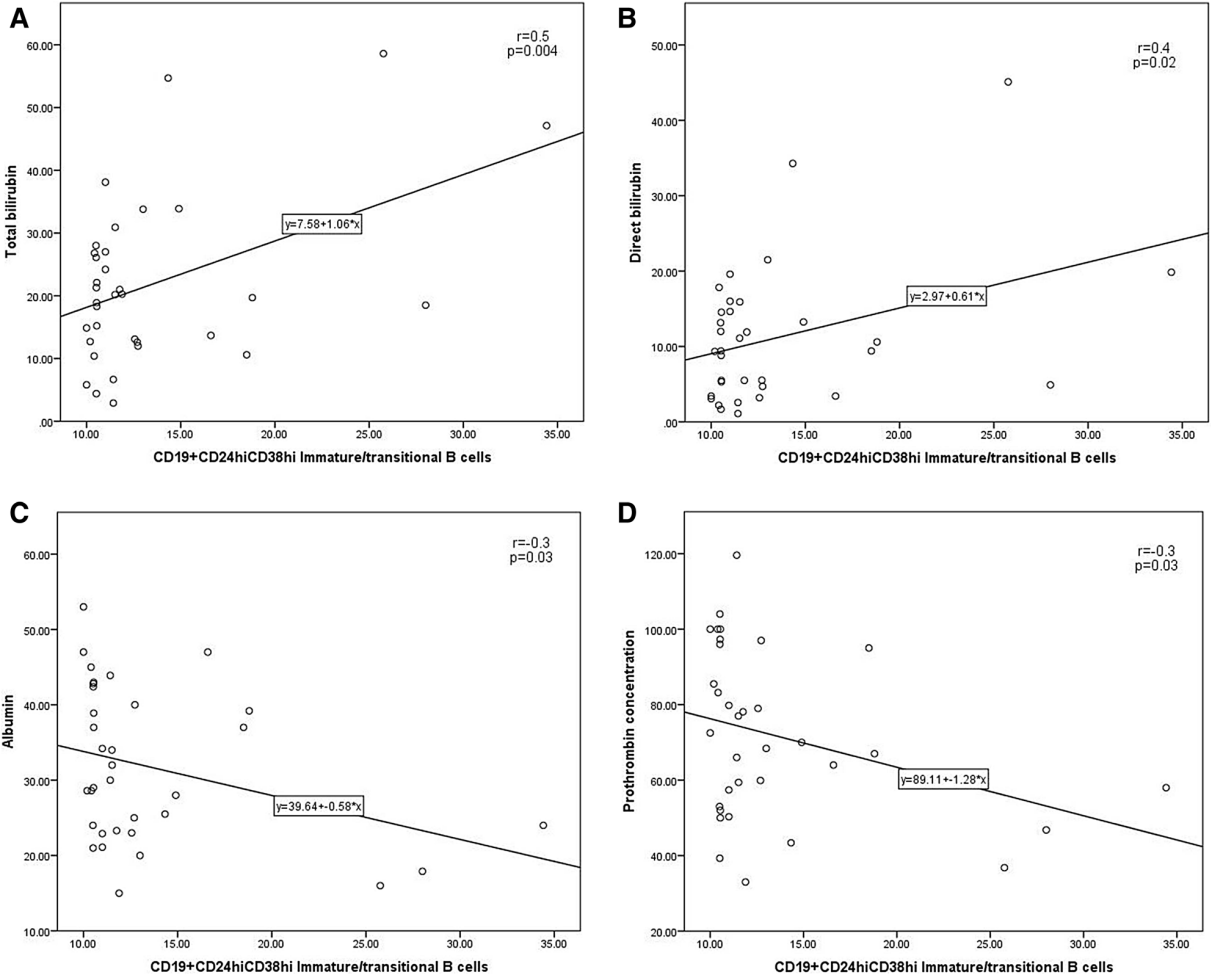
Correlations of CD19^+^CD24^hi^CD38^hi^ Immature/transitional B cells with (**A**) total bilirubin, (**B**) direct bilirubin, (**C**) Albumin and (**D**) prothrombin concentration in the diabetic CHC patients.

**Figure 3:**
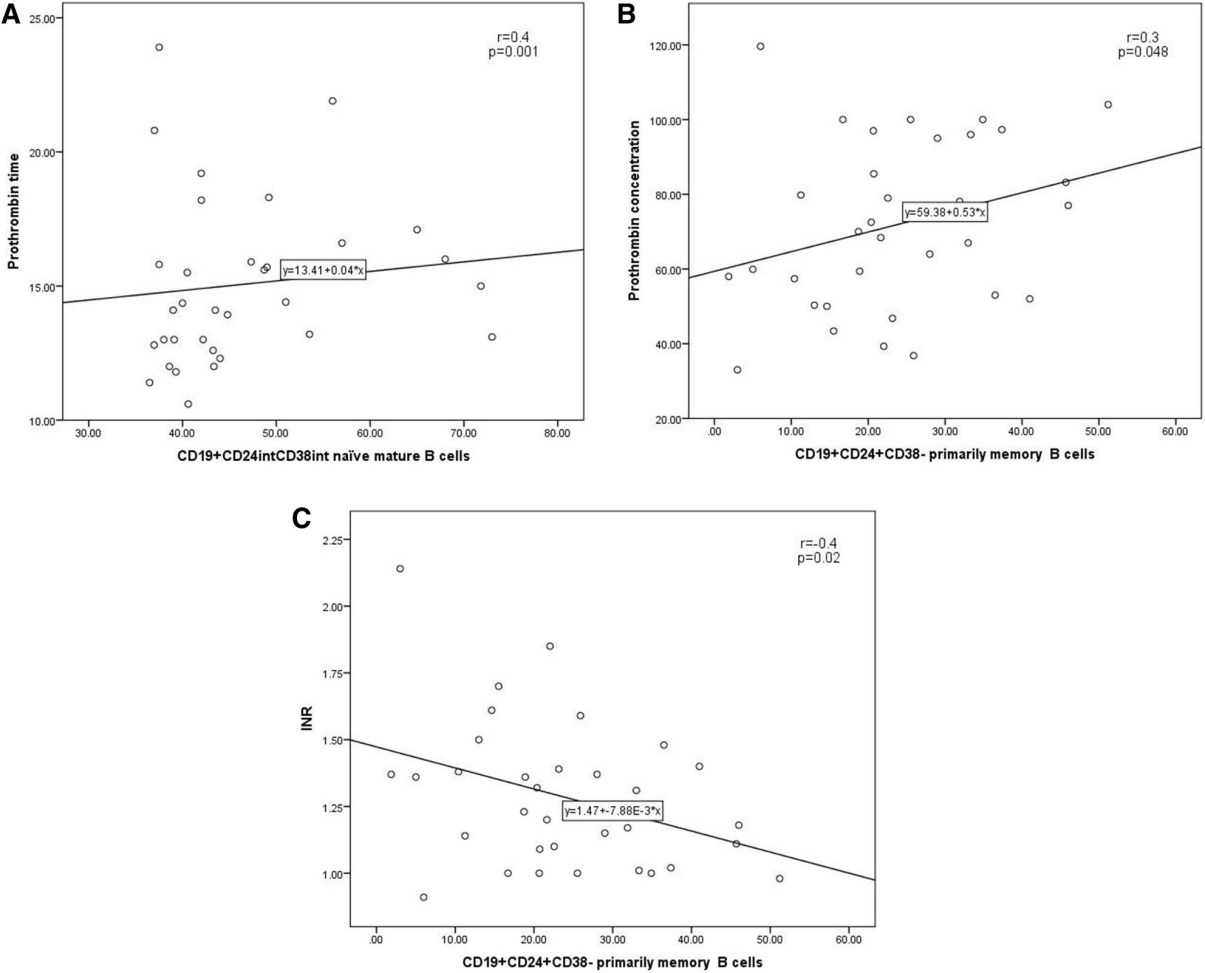
(**A**) Correlations of CD19^+^CD24^int^CD38^int^ naïve mature B cells with prothrombin time and correlations of CD19^+^CD24^+^CD38^−^ primarily memory B cells with (**B**) prothrombin concentration and (**C**) INR in the diabetic CHC patients.

**Figure 4:**
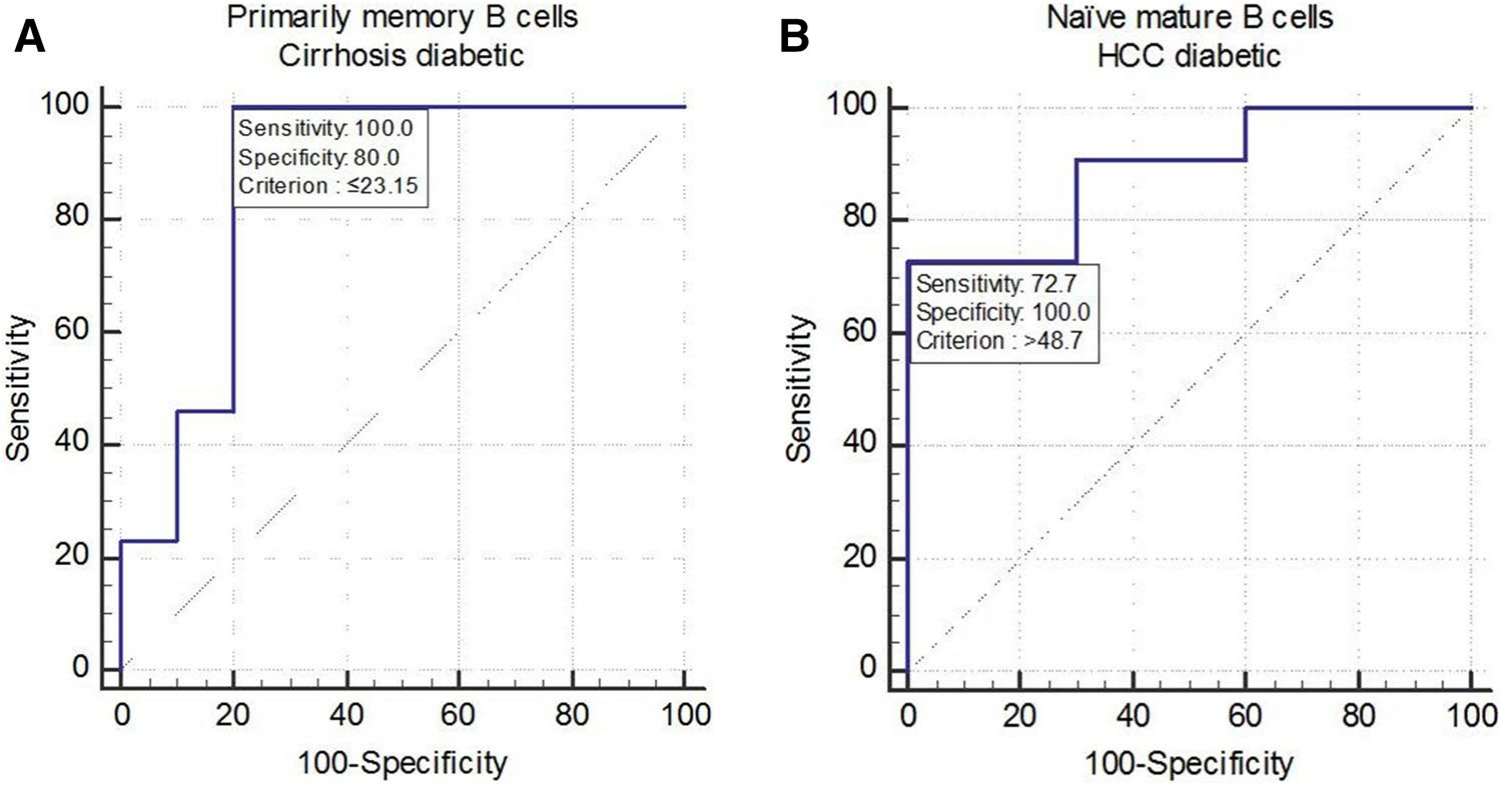
Receiver operating characteristic (ROC) curve of (**A**) CD19^+^CD24^+^CD38^−^ primarily memory B cells and (**B**) CD19^+^CD24^int^CD38^int^ naïve mature B cells, for prediction of liver cirrhosis and hepatocellular carcinoma, respectively, in diabetic chronic hepatitis C patients.

### Statistical analysis

The data were tested for normality using the Anderson–Darling test and for homogeneity of variances before further statistical analysis. Categorical variables were described by number and percent, whereas continuous variables were described by the mean and standard deviation or standard error (Mean, SD or SE). Between groups difference was investigated by the multivariate analysis of covariance (MANCOVA) with age and sex as covariates. A two-tailed *p* < 0.05 was considered statistically significant. Pearson correlation was applied to evaluate the association between variables. A receiver operating characteristic (ROC) curve was used to determine the cut-off value, sensitivity, specificity, and accuracy of each B cell subset in the prediction of cirrhosis and HCC. Statistical analysis was performed by the SPSS version 20.0 software (IBM, USA).

## Results

### Patients’ characteristics

The study encompassed 36 males (53.7%) and 31 females (46.3%). Their ages ranged from 31 to 67 years, with a mean age of 59.4 ± 9 years. Table 1 demonstrates the characteristics of the different patients’ groups. All patients had elevated levels of ALT and AST, with the highest levels observed in the diabetic CHC-HCC patients. Bilirubin levels increased in all groups, especially the cirrhotic patients.

**Table 1:** Characteristics of the different patients’ groups

Variable	Non-diabetic CHC n = 33			Diabetic CHC n = 34			Control (n = 27)
	CHC-N n = 10	CHC-LC n = 11	CHC-HCC n = 12	CHC-N n = 10	CHC-LC n = 13	CHC-HCC n = 11	
Demographic data							
Gender							
Male	3 (30%)	6 (54.5%)	10 (83.3%)	5 (50%)	5 (38.5%)	7 (63.6%)	15 (56%)
Female	7 (70%)	5 (45.5%)	2 (16.7%)	5 (50%)	8 (61.5%)	4 (36.4%)	12 (44%)
Age (years)	54.5 ± 12	62.8 ± 9	61.4 ± 5	57.4 ± 9	59.5 ± 8	59.7 ± 8	55 ± 0.7
Liver function tests							
ALT (IU/L)	*37.4* ± 19	50.2 ± 37	45.6 ± 42	*41.7* ± *21*	*54.3* ± 46	70.1 ± 71	9 ± 2
AST (IU/L)	35.9 ± 12	76.4 ± 58	71.4 ± 58	58.9 ± 49	79.7 ± 57	83.4 ± 75	8.5 ± 2
Albumin (g/dl)	3.9 ± .4	2.6 ± .8	3.1 ± .9	3.9 ± .7	2.3 ± .4	3.4 ± .1	4.1 ± 0.4
A/G ratio	1.1 ± 0.3	0.7 ± 0.2	0.9 ± 0.3	1.5 ± 0.8	0.7 ± 0.3	1 ± 0.4	2.7 ± 0.4
Total protein (g/dl)	7.7 ± .4	6.1 ± 2.3	6.6 ± 1.3	6.9. ± .7	6.4 ± .1	7.3 ± .9	7 ± 0.6
Total bilirubin (µmol/L)	13.3 ± 7	34.06 ± 32	20.04 ± 12	15.2 ± 7	27.3 ± 12	21.6 ± 15	5.7 ± 2
Direct bilirubin (µmol/L)	5.2 ± 4	14.1 ± 11	8.3 ± 6	7 ± 5	14 ± 8	11.6 ± 12	2.5 ± 1
Prothrombin time (sec)	12.1 ± 0.9	17.9 ± 4	16.1 ± 6	12.6 ± 1	16.1 ± 3	16.1 ± 2	12.8 ± 0.2
Prothrombin concentration (%)	96.3 ± 11.3	55.4 ± 23.7	68.2 ± 20	96.2 ± 13	60 ± 16	63.3 ± 17	94.6 ± 5
INR	1 ± 0.05	1.6 ± 0.4	1.4 ± 0.6	1.1 ± 0.1	1.4 ± 0.3	1.4 ± 0.2	1 ± 0.1
HCV-RNA copy number							
PCR (copies/ml)	1.6 × 10^6^ ± 2 × 10^6^	0.8 × 10^6^ ± 10^6^	1 × 10^6^ ± 10^6^	7 × 10^6^ ± 16 × 10^6^	1.3 × 10^6^ ± 2 × 10^6^	0.7 × 10^6^ ± 10^6^	Nil
Plasma glucose level							
Fasting plasma glucose (mg/dl)	90 ± 12	96 ± 12	89 ± 14	247 ± 113	236 ± 66	210 ± 58	86.3 ± 8

*CHC-N* chronic hepatitis C with no cirrhosis or carcinoma, *CHC-LC* liver cirrhosis, *CHC-HCC* hepatocellular carcinoma, *AST* Aspartate transaminase, *ALT* Alanine transaminase**,** *INR* International Normalized Ratio**,** *A/G* Albumin /Globulin ratio.

Results displayed as mean  ±  standard deviation (SD), *Result displayed as a number (percent from the corresponding group).

Similarly, the lowest levels of total protein, albumin, and A/G ratio were detected in cirrhotic patients. Marked affection of prothrombin time and concentration and international normalized ratio (INR) was seen in the CHC-LC and CHC-HCC patients. The diabetic CHC-N patients have shown the highest HCV load among the studied groups. The most elevated fasting plasma glucose (FPG) level among the studied groups was also observed in the diabetic CHC-N patients. No significant differences were observed between the diabetic (n = 33) and the non-diabetic (n = 34) patients in any measured laboratory parameters. Of all HCC patients, 13 had Child–Pugh score A, eight had score B, and one had score C.

### Analysis of CD19^+^ B cell subsets in chronic hepatitis C patients with respect to T2D:

As presented in Table 2, regardless of the degree of liver affection, the percentages of CD19^+^CD24^hi^CD38^hi^ Immature/transitional (from CD19^**+**^ B cells) in both the diabetic and the non-diabetic groups were higher than that in the healthy controls, but this difference was only significant in the diabetic group (mean, 13.6 ± 1, 10.7 ± 1 vs. 7.8 ± 1, *p* = 0.001, *p* = 0.1, respectively). They were also higher in diabetic patients compared with the non-diabetic patients (*p* = 0.03). Although the highest levels of CD19^+^CD24^int^CD38^int^ naïve mature B cells (from CD19^+^ B cells) were detected in diabetic patients, no statistically significant differences were observed among the three groups.

**Table 2:** CD19^+^ B cell subsets in chronic hepatitis C patients with respect to T2D.

B cell subsets (%)	Control n = 27	Diabetic CHC n = 34	Non-diabetic CHC n = 33	*p *values		
				*p1*	*p2*	*p3*
CD19^+^CD24^hi^CD38^hi^ B cells	7.8 ± 1	13.6 ± 1	10.7 ± 1	**0.001**	0.1	**0.03**
CD19^+^CD24^int^CD38^int^ B cells	42.7 ± 2	46.9 ± 1	42.9 ± 2	0.2	0.97	0.08
CD19^+^CD24^+^CD38^−^B cells	14.3 ± 3	23.8 ± 2	30.2 ± 2	**0.02**	** < 0.0001**	**0.04**
CD19^+^CD24^−^CD38^+^ B cells	1 ± 0.2	1.7 ± 0.1	1.8 ± 0.1	**0.007**	**0.003**	0.6
CD19^+^CD24^−^CD38^−^ B cells	32.2 ± 4	14.3 ± 3	14 ± 3	** < 0.0001**	** < 0.0001**	0.96

(%) the percentage was calculated from CD19^+^ B cells.

Results expressed as mean  ±  standard error.

Multivariate analysis of covariance (MANCOVA) with age and sex as covariates, Significant *p*-value is < 0.05 (bold).

*p*1: Control vs. Diabetic, *p*2: Control vs. Non-diabetic, *p*3 Diabetic vs. Non-diabetic patients.

The percentages of CD19^+^CD24^+^CD38^−^ primarily memory B cell subset (from CD19^+^ B cells) in both the diabetic and the non-diabetic groups were considerably raised than that in the healthy controls (mean, 23.8 ± 2, 30.2 ± 2 vs. 14.3 ± 3, *p* = 0.02, *p* < 0.0001, respectively). However, the level of this subset of B cells was considerably lower in diabetic patients when compared with non-diabetic patients (*p* = 0.04). On the contrary, a remarkable decrease was observed in the level of CD19^+^CD24^**−**^CD38^−^ new memory B cells in both the diabetic and non-diabetic groups than the healthy controls (mean, 14.3 ± 3, 14 ± 3 vs. 32.2 ± 4, *p* < 0.0001 for both), yet with no significant differences between the diabetic and non-diabetic groups (*p* = 0.96).

On the other hand, levels of CD19^+^CD24^−^CD38^+^ plasmablasts were significantly elevated in both the diabetic and non-diabetic patients than healthy controls (mean, 1.7 ± 0.1, 1.8 ± 0.1 vs. 1 ± 0.2, *p* = 0.007, *p* = 0.003, respectively), with no difference between the diabetic and non-diabetic patients (*p* = 0.6).

### Characterization of CD19^+^ B cell population in chronic hepatitis C patients with respect to the degree of liver affection

As shown in Table 3, irrespective of the presence of diabetes, the percentage of CD19^+^CD24^hi^CD38^hi^ Immature/transitional B cells (from CD19^+^ B cells) was elevated in the CHC-N, CHC-LC and CHC-HCC patients than the healthy controls (mean, 11.2 ± 1, 13.2 ± 1, 12.5 ± 1 vs. 7.7 ± 2, *p* = 0.07, *p* = 0.007, *p* = 0.02, respectively). Meanwhile, no statistically considerable differences were observed among the three groups of patients. On the other hand, the percentage of CD19^+^CD24^int^CD38^int^ naïve mature B cells (from CD19^+^ B cells) was elevated in the CHC-HCC patients in comparison with both the control (mean, 48.4 ± 2 vs. 42.5 ± 2, *p* = 0.07) and the CHC-N group (mean, 48.4 ± 2 vs. 41.4 ± 2, *p* = 0.01).

**Table 3:** CD19^+^ B cell subsets in chronic hepatitis C patients with respect to the degree of liver affection.

B cell subsets (%)	Control n = 27	CHC-N n = 20	CHC-LC n = 24	CHC-HCC n = 23	*p*-values					
					*p1*	*p2*	*p3*	*p4*	*p5*	*p6*
CD19^+^CD24^hi^CD38^hi^ B cells	7.7 ± 2	11.2 ± 1	13.2 ± 1	12.5 ± 1	0.07	**0.007**	**0.02**	0.2	0.5	0.7
CD19^+^CD24^int^CD38^int^ B cells	42.5 ± 2	41.4 ± 2	45.6 ± 2	48.4 ± 2	0.7	0.3	0.07	0.1	**0.01**	0.3
CD19^+^CD24^+^CD38^−^ B cells	14.6 ± 3	29.4 ± 3	21.5 ± 3	29 ± 3	**0.001**	0.1	**0.002**	**0.04**	0.9	**0.04**
CD19^+^CD24^−^CD38^+^ B cells	1 ± 0.2	1.7 ± 0.1	1.8 ± 0.1	1.8 ± 0.2	**0.01**	**0.006**	**0.008**	0.7	0.7	0.9
CD19^+^CD24^−^CD38^−^ B cells	32 ± 4	15 ± 3	17.3 ± 3	10 ± 3	**0.001**	**0.005**	** < 0.0001**	0.6	0.3	0.1

(%) the percentage was calculated from CD19^+^ B cells.

*CHC-N* chronic hepatitis C with no cirrhosis or hepatocellular carcinoma, *CHC-LC* chronic hepatitis C with liver cirrhosis, *CHC-HCC* chronic hepatitis C with hepatocellular carcinoma.

Results expressed as mean  ±  standard error.

Multivariate analysis of covariance (MANCOVA) with age and sex as covariates Significant *p-*value is < 0.05 (bold).

*p*1: CHC-N vs. Control *p*2: CHC-LC vs. Control *p*3: CHC-HCC vs. Control.

*p*4: CHC-N vs. CHC-LC *p*5: CHC-N vs. CHC-HCC *p*6: CHC-LC vs. CHC-HCC.

In the same way, an elevation of the frequency of CD19^+^CD24^+^CD38^−^ primarily memory B cell subset (from CD19^+^ B cells) was observed in the CHC-N, CHC-LC, and CHC-HCC patients compared with the healthy controls (mean, 29.4 ± 3, 21.5 ± 3, 29 ± 3 vs. 14.6 ± 3, *p* = 0.001, *p* = 0.1, *p* = 0.002, respectively), but with no significant difference between CHC-LC patients and control group. Moreover, its level was raised in both the CHC-N and CHC-HCC patients than in the CHC-LC patients (*p* = 0.04, for both groups).

On the contrary, the frequency of CD19^+^CD24^**−**^CD38^−^ new memory B cells (from CD19^+^ B cells) decreased in the CHC-N, CHC-LC, and CHC-HCC patients compared with the healthy controls (mean, 15 ± 3, 17.3 ± 3, 10 ± 3 vs. 32 ± 4, *p* = 0.001, *p* = 0.005, *p* < 0.0001, respectively) and was somewhat lower in both the CHC-N and CHC-HCC patients than in the CHC-LC patients.

Also, the frequencies of CD19^+^CD24^−^CD38^+^ plasmablasts in the CHC-N, CHC-LC, and CHC-HCC groups were higher than that in the control group (mean, 1.7 ± 0.1, 1.8 ± 0.1, 1.8 ± 0.2 vs. 1 ± 0.2, *p* = 0.01, *p* = 0.006, *p* = 0.008, respectively), with no statistically significant differences among the three groups of patients.

### Analysis of the CD19^+^ B cell subsets in CHC patients with different degrees of liver affection with respect to T2D

Results are shown in Table 4.

**Table 4:** CD19^+^ B cell subsets in chronic hepatitis C patients with different degrees of liver affection in relation to diabetes mellitus.

Diabetic										
B cell subsets (%)	Control n = 27	CHC-N n = 10	CHC-LC n = 13	CHC-HCC n = 11	*p* values					
					*p1*	*p2*	*p3*	*p4*	*p5*	*p6*
CD19^+^CD24^hi^CD38^hi^ B cells	7.7 ± 2	12.3 ± 2	15 ± 1	13 ± 2	**0. 046**	**0.001**	**0.02**	0.2	0.7	0.4
CD19^+^CD24^int^CD38^int^ B cells	42.7 ± 2	40.6 ± 3	47.3 ± 2	52.5 ± 3	0.6	0.2	**0.007**	0.05	**0.002**	0.1
CD19^+^CD24^+^CD38^−^ B cells	14.3 ± 3	31.2 ± 3	14.6 ± 3	27.9 ± 3	** < 0.0001**	0.9	**0.004**	** < 0.0001**	0.5	**0.004**
CD19^+^CD24^−^CD38^+^ B cells	1 ± 0.2	1.7 ± 0.2	1.9 ± 0.2	1.6 ± 0.2	0.06	**0.007**	0.07	0.5	0.9	0.4
CD19^+^CD24^−^CD38^−^ B cells	32.3 ± 4	13 ± 4	19.8 ± 4	8.6 ± 4	**0.002**	**0.03**	** < 0.0001**	0.3	0.5	0.06

Non-diabetic										
B cell subsets (%)	Control n = 27	CHC-N n = 10	CHC-LC n = 11	CHC-HCC n = 12	*p v*alues					
					*p1*	*p2*	*p3*	*p4*	*p5*	*p6*
CD19^+^CD24^hi^CD38^hi^ B cells	7.7 ± 2	10.4 ± 2	10 ± 2	11.6 ± 2	0.2	0.3	0.1	0.9	0.6	0.6
CD19^+^CD24^int^CD38^int^ B cells	42.7 ± 2	42.7 ± 3	42.6 ± 3	43.4 ± 3	0.98	0.99	0.9	0.98	0.9	0.9
CD19^+^CD24^+^CD38^−^ B cells	14.3 ± 3	26.6 ± 3	33.4 ± 4	31.6 ± 4	**0.009**	** < 0.0001**	**0.001**	0.2	0.3	0.7
CD19^+^CD24^−^CD38^+^ B cells	1 ± 0.2	1.8 ± 0.2	1.9 ± 0.2	1.9 ± 0.2	**0.03**	**0.05**	**0.007**	0.9	0.6	0.5
CD19^+^CD24^−^CD38^−^ B cells	32.3 ± 4	17.5 ± 4	12.8 ± 5	11 ± 5	**0.02**	**0.005**	**0.001**	0.5	0.3	0.8

(%) the percentage was calculated from CD19^+^ B cells.

*CHC-N* chronic hepatitis C with no cirrhosis or hepatocellular carcinoma, *CHC-LC* chronic hepatitis C with liver cirrhosis, *CHC-HCC* chronic hepatitis C with hepatocellular carcinoma.

Results expressed as mean  ±  standard error.

Multivariate analysis of covariance (MANCOVA) with age and sex as covariates, Significant *p*-value is < 0.05 (bold).

*p*1: CHC-N vs. Control *p*2: CHC-LC vs. Control *p*3: CHC-HCC vs. Control.

*p*4: CHC-N vs. CHC-LC *p*5: CHC-N vs. CHC-HCC *p*6: CHC-LC vs. CHC-HCC.

#### Analysis of CD19^+^CD24^hi^CD38^hi^ Immature/transitional B cells

In the diabetic patients alone, the frequencies of Immature/transitional B cells in the three diabetic groups (CHC-N, CHC-LC and CHC-HCC) were significantly higher than the controls (mean, 12.3 ± 2, 15 ± 1, 13 ± 2 vs. 7.7 ± 2, *p* = 0.046, *p* = 0.001, *p* = 0.02, respectively). The highest level of Immature/transitional B cells was observed in cirrhotic patients but with no significant differences from the other two groups of patients. In the meantime, no significant differences were detected among the three non-diabetic groups.

#### Analysis of CD19^+^CD24^int^CD38^int^ naïve mature B cells

In the diabetic groups, the highest level of CD19^+^CD24^int^CD38^int^ B cells was seen in the CHC-HCC patients (52.5 ± 3), in comparison with the controls (*p* = 0.007) and the CHC-N group (*p* = 0.002). Meanwhile, no significant differences were observed in the frequencies of CD19^+^CD24^int^CD38^int^ B cells among the three non-diabetic groups and the control group.

#### Analysis of CD19^+^CD24^+^CD38^−^ primarily memory B cells

In the diabetic patients, the frequency of CD19^+^CD24^+^CD38^−^ B cells notably increased in the CHC-N and CHC-HCC patients compared with the controls (mean, 31.2 ± 3, 27.9 ± 3 vs. 14.3 ± 3, *p* < 0.0001, *p* = 0.004, respectively) and the CHC-LC group (*p* < 0.0001, *p* = 0.004, respectively). In contrast, in the non-diabetic patients, the levels of CD19^+^CD24^+^CD38^−^ primarily memory B cells were significantly high in the three patients^’^ groups (CHC-N, CHC-LC, and CHC-HCC) in comparison with the controls (mean, 26.6 ± 3, 33.4 ± 4, 31.6 ± 4 vs. 14.3 ± 3, *p* = 0.009, *p* < 0.0001, *p* = 0.001, respectively), with no significant differences among the three non-diabetic groups of patients.

#### Analysis of CD19^+^CD24^−^CD38^−^ new memory B cells

Level of CD19^+^CD24^−^CD38^−^ new memory B cells (from CD19^+^ B cells) significantly decreased in the CHC-N, CHC-LC and CHC-HCC groups compared with the healthy controls in both the the diabetic patients ((*p* = 0.002, *p* = 0.03 and *p* < 0.0001, respectively), and the non-diabetic patients (*p* = 0.02, *p* = 0.005 and *p* = 0.001, respectively). But no significant differences were observed between the patients’ groups.

#### Analysis of CD19^+^CD24^−^CD38^+^ plasmablasts

In the diabetic groups, this B cell subset was only presenting higher levels in the CHC-LC group than the healthy controls (*p* = 0.007). On the contrary, the frequencies of CD19^+^CD24^−^CD38^+^ plasmablasts raised in the three non-diabetic groups (CHC-N, CHC-LC and CHC-HCC) compared with the controls (mean, 1.8 ± 0.2, 1.9 ± 0.2, 1.9 ± 0.2 vs. 1 ± 0.2, *p* = 0.03, *p* = 0.05, *p* = 0.007, respectively), with no significant differences among the three non-diabetic groups of patients.

### Evaluation of the associations between the different B cell subsets and the laboratory findings with respect to T2D

Several significant associations were detected, especially among the diabetic patients.

#### CD19^+^CD24^hi^CD38^hi^ Immature/transitional B cells

As shown in Fig. 2, in the diabetic patients, immature/transitional B cells revealed direct associations with both total and direct bilirubin levels (r = 0.5, *p* = 0.004 and r = 0.4, *p* = 0.02, respectively) and inverse relations with both albumin and prothrombin concentration (r = −0.3, *p* = 0.03 and r = −0.3, *p* = 0.03, respectively). Additionally, these cells had direct relation with the Child–Pugh score in the non-diabetic and diabetic CHC-HCC patients (r = 0.7, *p* = 0.03 and r = 0.5, *p* = 0.04, respectively). Moreover, immature/transitional B cells were the only cells showing direct relation with age (r = 0.3, *p* = 0.04) in the diabetic patients alone.

#### CD19^+^CD24^int^CD38^int^ naïve mature B cells and CD19^+^CD24^−^CD38^+^ plasmablasts

As shown in Fig. 3, an association was observed between CD19^+^CD24^int^CD38^int^ B cells and prothrombin time (r = 0.4, *p* = 0.001). On the other hand, no significant associations were observed in the diabetic and non-diabetic groups of patients between CD19^+^CD24^−^CD38^+^ plasmablasts and any measured laboratory parameters.

#### CD19^+^CD24^+^CD38^−^ primarily memory and CD19^+^CD24^−^CD38^−^ new memory B cells

As shown in Fig. 3, CD19^+^CD24^+^CD38^−^ B cells have shown direct association with prothrombin concentration and albumin level (r = 0.3, *p* = 0.048 and r = 0.3, *p* = 0.05, respectively) and inverse relation with INR (r = -0.4, *p* = 0.02). Conversely, no significant correlations were detected between the level of CD19^+^CD24^−^CD38^−^ new memory B cells and any of the laboratory findings in both the diabetic and non-diabetic patients.

### Evaluation of the different B cell subsets as probable predictors of cirrhosis and HCC in CHC patients

The Receiver Operating Characteristic (ROC) curve has been used to evaluate the accuracy of using the blood levels of different B cell subsets as potential predictors of developing LC and HCC in CHC patients. None of the B cell subsets showed good accuracy in predicting either the development of LC or HCC in the non-diabetic CHC patients.

On the other hand, in diabetic patients, among all B cell subsets, CD19^+^CD24^+^CD38^−^ primarily memory B cells showed good accuracy in predicting LC [accuracy = 90%, area under the curve (AUC) = 0.9, *p* > 0.0001]. The best cut-off for the prediction of LC was ≤ 23.2 (% from CD19^+^ B cells) with a sensitivity of 100% and specificity of 80% Fig. 4. Furthermore, Immature/transitional B cells showed moderate accuracy in prediction of LC [accuracy = 77%, AUC = 0.72, *p* = 0.03]. The best cut off for the prediction of LC was > 10.5 (% from CD19^+^ B cells) with a sensitivity of 85% and specificity of 70%.

CD19^+^CD24^int^CD38^int^ naïve mature B cells were the only B cell subset showing good prognostic accuracy in the prediction of HCC [accuracy = 86.4%, AUC = 0.9, *p* = 0.006]. The best cut-off for the prediction of HCC was < 48.7 (% from CD19^+^ B cells) with a sensitivity of 73% and specificity of 100%, Fig. 4.

## Discussion

Little is known about the changes in B cell subsets in T2D patients^28^ and CHC patients^25,26,29^, and to date, seemingly no one has addressed the relations of B cell subsets with liver disease progression in the diabetic CHC patients. For this purpose, B cell subsets were analyzed in the peripheral blood of 67 CHC patients to avoid invasive techniques which may reflect, at least qualitatively, subset distribution in tissues such as bone marrow, lymph nodes, and liver^30^. To verify the relation of the different B cell subsets with liver disease progression in CHC diabetic patients, we first compared the levels of the B cell subsets between the diabetic and non-diabetic patients regardless of the severity of liver affection. Then we compared CHC-N, CHC-LC, and CHC-HCC patients irrespective of the presence of diabetes. Finally, we compared the levels of the different B cell subsets between CHC-N, CHC-LC, and CHC-HCC patients in the diabetic and the non-diabetic groups.

Apparently, CD19^+^CD24^hi^CD38^hi^ Immature/transitional B cells have a relation with CHC disease pathogenesis with impacts on liver functions. That was apparent where immature/transitional B cells were significantly higher in the CHC-LC, and CHC-HCC compared with the control group. This effect seems more pronounced in diabetic patients, where the level of this subset was considerably elevated in the three groups of diabetic patients (CHC-N, CHC-LC, and CHC-HCC). In contrast, in the non-diabetic patients, although it raised in the CHC-HCC patients and positively correlated with the Child–Pugh score in those patients, this rise was not significant. Besides, only in diabetic patients, Immature/transitional B cells revealed positive correlations with the levels of total bilirubin, direct bilirubin and Child–Pugh score. In addition, to their inverse relations with serum albumin and prothrombin concentration. Moreover, by the ROC curve analysis, Immature/transitional B cells showed moderate accuracy in predicting CHC progression to LC. Our results open new avenues for investigating this subset of B cells as a potential target for immunotherapy in those patients.

In general, data regarding B cell subsets in T2D are deficient, and most of the studies on T1D are on mice. An earlier study^31^ didn’t find significant discrepancies in the levels of transitional, mature naïve, memory, or plasmablast subsets between T1D subjects and controls. Conversely, they reported a reduced relative frequency of CD24^hi^CD38^hi^ B cells in T1D, which might be the regulatory B cell population. Similarly, other studies stated that a lower level of IL-10 producing Bregs is associated with insulin intolerance^32^ and T1D^33^. This inconsistency with our findings might be ascribed to the differences in the study populations since our patients have T2D and are infected by HCV. Whether this B cell subset is disturbed or not in T2D requires further confirmatory research.

Sugalski and colleagues^25^ described an increased frequency of immature transitional B cell subset in the HCV infected patients. They proposed that this may be due to tissue redistribution, enhanced production, or repressed cell death. They found a direct relationship between serum BAFF level, a factor essential for late transitional B cell survival and differentiation^34^, and the frequency of immature transitional B cell. Likewise, they observed increased expression of Ki67, a cellular marker of proliferation, by immature transitional B cells, which may be induced by HCV.

Previously, Bregs were found abundant in the tumor microenvironment, and that aided the progression of HCC. Bregs suppress the immune responses against HCC primarily via the CD40/CD40L mediated production of IL-10 and TGF-β, which down-regulate TNF-α that is critical for halting tumor progression^35,36^. Bregs may suppress the T cell-mediated anticancer immune response in the tumor microenvironment by inducing the CD4^+^ T cell conversion into Tregs, promoting tumor progression and metastasis^37^. Bregs promote dendritic cells to switch the secretion of IL-12 to IL-4, thereby affecting the Th1/Th2 balance, favoring the induction of a Th2 polarization^38^.

The CD19^+^CD24^int^CD38^int^ naïve mature B cells, in the present study, were only significantly high in diabetic CHC-HCC patients in comparison with CHC-N and CHC-LC patients and the healthy controls. Also, this B cell subset showed association with the prothrombin time. Likewise, the analysis of the ROC curve showed that this cell population has good accuracy in the prediction of HCC in diabetic CHC patients. The relation of this B cell subset with the pathogenesis and progression of HCC in diabetic patients needs detailed prospect studies.

The CD19^+^CD24^+^CD38^−^ primarily memory B cells were generally less in the diabetic than the non-diabetic groups irrespective of the liver disease stage. Interestingly in the diabetic patients alone, it was lower in the CHC-LC than the CHC-N and CHC-HCC groups. Furthermore, only in diabetic patients, lower levels of primarily memory B cells were associated with lower serum albumin and prothrombin concentration, and higher INR. The ROC curve analysis also showed good predictive accuracy of the levels of primarily memory B cells for the development of LC in the diabetic CHC patients alone and was even more accurate than the immature/transitional B cells. The opposite was observed in Level of CD19^+^CD24^−^CD38^−^ new memory B cells where their level decreased in the CHC-N, CHC-LC and CHC-HCC diabetic and non-diabetic patients compared with the control.

Fournillier and colleagues^39^ showed that the levels of naïve and memory B cells and their signaling via BCR stimulation were normal in HCV-infected individuals. Another study^26^ inconsistently reported that memory but not naïve B cells showed a lower level in patients with cirrhosis compared with patients with little or no fibrosis, which may be attributed to enhanced apoptosis^40^ or migration to the liver^41^.

Despite the B cell overactivity, hypergammaglobulinemia, and increased liability to B lymphocyte proliferative disorders observed in CHC patients^20–24^, the vast majority of the anti-HCV antibodies lack the convenient viral neutralizing functions^42^. Unlike naïve B cells, which are entirely in need of BCR signaling, memory B cells may be activated and rapidly differentiate to plasma cells^43–45^ when they are signaled by the armed helper T cell even without BCR triggering^30,43,44^. Lower levels of memory B cells and higher levels of plasmablasts were observed in obese patients with T2D compared with non-obese diabetic patients^28^. This may be because when memory B cells challenge a distinct antigen during inflammation, they proliferate and differentiate into antibody-producing plasmablasts^46^. Thus, the plasmablasts population is a frequently used marker for B cell activation in inflammation^47^.

In the current study, regardless of the presence of diabetes, significant elevation of the CD19^+^CD24^−^CD38^+^ plasmablasts was seen in the CHC-N, CHC-LC, and CHC-HCC groups of patients. Furthermore, the highest level of plasmablasts was detected in the CHC-LC diabetic patients compared to the other patients’ groups. These findings collectively may support the previously mentioned theory of primarily memory B cell activation and differentiation to antibody-producing plasmablasts^28^, which entails more inflammation in the diabetic patients that will ultimately lead to liver damage and fibrosis with a deterioration of the liver functions.

### Strengths and limitations

This study was the first to analyze the different B cell subsets in CHC patients with T2D. Overall our results demonstrate a strong association between B cell subsets and liver disease progression in those patients. Thus the future research on these subsets might prove their value in pathogenesis, immunotherapy, and as predictors of disease progression. Still, this study has some potential limitations; none of the tested B cell subsets has shown noteworthy relation with the fasting plasma sugar level. This may be because most of the patients had controlled sugar levels at the time of sample collection. Significant correlations might have become evident if HbA1C was measured, which reflects the average level of glucose during the last 2–3 months^48^. Another limitation was the small sample size. To yield more reliable estimates, the study needs to be done on a larger cohort of patients. Also, for a more comprehensive evaluation of B cell subsets, other fundamental markers and cytokines should be assessed including, CD27 and IL10. Lack of studies based on similar B cell subsetting panels made it difficult to draw consistent conclusions.

## Conclusion

Immature/transitional B cells increase remarkably in diabetic CHC patients and might have a role in liver disease progression. Memory and Naïve B cells are good potential predictors of LC and HCC in diabetic CHC patients, respectively. Further studies are needed to investigate the role of the CD19^+^CD24^−^CD38^−^ new memory B cells in disease progression in CHC patients.
